# The association between different opioid doses and the survival of advanced cancer patients receiving palliative care

**DOI:** 10.1186/s12904-016-0169-5

**Published:** 2016-11-21

**Authors:** Anon Sathornviriyapong, Kittiphon Nagaviroj, Thunyarat Anothaisintawee

**Affiliations:** Department of Family Medicine, Faculty of Medicine, Ramathibodi Hospital, Mahidol University, 270 Rama VI Street, Rajthevi, Bangkok, 10400 Thailand

## Abstract

**Background:**

Concerns that opioids may hasten death can be a cause of the physicians’ reluctance to prescribe opioids, leading to inadequate symptom palliation. Our aim was to find if there was an association between different opioid doses and the survival of the cancer patients that participated in our palliative care program.

**Methods:**

A retrospective study was conducted at Ramathibodi Hospital, Bangkok between January 2013 and December 2015. All of the cancer patients that were referred to palliative care teams by their primary physicians were included in the study. The study data included the patients’ demographics, disease status, comorbidities, functional status, type of services, cancer treatments, date of consultation, and the date of the patient’s death or last follow-up. The information concerning opioid use was collected by reviewing the medical records and this was converted to an oral morphine equivalent (OME), following a standard ratio. The time-varying covariate in the Cox regression analysis was applied in order to determine the association between different doses of opioids and patient survival.

**Results:**

A total of 317 cancer patients were included in the study. The median (IQR) of the OME among our patients was 6.43 mg/day (0.53, 27.36). The univariate Cox regression analysis did not show any association between different opioid doses (OME ≤ 30 mg/day and > 30 mg/day) and the patients’ survival (*p* = 0.52). The PPS levels (*p* < 0.01), palliative care clinic visits (HR 0.32, 95%CI 0.24–0.43), home visits (HR 0.75, 95%CI 0.57–0.99), chemotherapy (HR 0.32, 95%CI 0.22–0.46), and radiotherapy (HR 0.53, 95%CI 0.36–0.78) were identified as factors that increased the probability of survival.

**Conclusions:**

Our study has demonstrated that different opioid doses in advanced cancer patients are not associated with shortened survival period.

## Background

Opioids have been recommended by the WHO for the first-line treatment of moderate to severe cancer pain and the use of strong opioids for dyspnea management has been considered an efficacious and safe treatment [1, 2]. Despite the availability of the drugs, inadequate pain management has been found among cancer patients [3, 4]. One of the most frequent causes of undertreatment is misconceptions about opioids [5–8].

There are still ongoing fears surrounding the use of opioid analgesics among patients, families, and health care professionals. A qualitative study revealed that many patients and families believed that opioids should be only used in terminal cases and at the end of life, and that opioid use may be associated with premature death [5]. Another study that attempted to identify the barriers to cancer pain management in Taiwan demonstrated that informed family caregivers of advanced cancer patients had concerns about reporting pain and administering opioids, particularly as they related to disease progression and possible side effects [7]. Additionally, previous studies on the physician’s attitude toward prescribing opioids for dyspnea showed that the most frequently-reported barriers regarding the prescription of opioids were the resistance of the patient, fear of potential adverse effects, and fear of respiratory depression [6, 8]. A study in the Netherlands on the perceptions of physicians concerning opioid use and the survival of the patient showed that physicians more often took hastening death into account when they gave higher doses of opioids when the patient experienced more severe symptoms and with female patients [9].

With regard to the concerns of the potential adverse events related to opioid use, the current evidence regarding the effect of opioids on the patient’s survival is still conflicting. Some studies have indicated a potential association of increased survival with higher doses of opioids or increases in opioid doses in the last days of life [10–14]. Some studies on the other hand did not show any significant survival difference between those that were taking opioids and those that were not [15–17]. In other studies, higher opioid doses or increasing doses of opioids were reported to be associated with shorter survival, although some of these did not examine the effect of opioids on survival as a primary endpoint [18–20]. Several retrospective studies have suggested that opioid use might promote tumor progression and as a result negatively impact the survival of patients with advanced cancer [21–23]. Additionally, opioid doses have been seen to be correlated with low testosterone, and hypogonadal males were seen to have a shorter survival compared with those that were eugonadal [24]. The analysis from a large chemotherapy RCT showed that opioid use was independently associated with shorter survival [25]. Regarding two recent systematic reviews on the association between systemic opioid analgesia and survival among cancer patients [26, 27], the results showed that there was no clear association between opioid doses or increasing doses of opioids and survival; other studies showed that opioids might be associated with decreased survival, while others suggested that opioids improved survival or had no effect.

The Department of Family Medicine at Ramathibodi Hospital, Mahidol University, had a project on the development of palliative care for people in the Bangkok metropolitan area beginning in 2010. The project is aimed at the integration of palliative care services into mainstream medicine in Thailand by providing a variety of services, including inpatient consultation, a palliative care clinic, telephone consultation, as well as providing home-based palliative care. Around 700 patients and their families participated in this project from January 2010 to December 2015. Among these patients, 90% were diagnosed with cancer. The aim of this study was to find if there was an association between different opioid doses and the survival of the cancer patients that participated in our palliative care program.

## Methods

This retrospective cohort study was conducted in the Department of Family Medicine at Ramathibodi Hospital, Bangkok, Thailand, between January 2013 and December 2015. All of the patients with a cancer diagnosis that were referred to palliative care teams by their primary physicians, e.g., oncologists, surgeons or internists (both inpatient and outpatient consultation), were included into the study. The time from the first palliative consultation to the study endpoints was estimated for each subject. The endpoints of patient follow up were death, referral to other catchment areas, or the end of the study period. The study’s participants were censored, if they were referred to other catchment areas or were still alive at the end of study period (December 31^th^, 2015). The primary outcome was all-cause mortality.

### Data collection

The study data were collected using a standardized data record form. The data set was comprised of the patient’s demographics (age, gender, marital status), disease status (primary tumor sites, presence of metastases), comorbidities assessed by using the Charlson Comorbidity Index(CCI) [28], functional status assessed using the Palliative Performance Scale Adult Suandok, which was translated into Thai from the Palliative Performance Scale (PPSv2) [29], types of palliative care service, concurrent cancer treatments, date of patient consultation with the palliative care service, and the date of death or the patient’s last follow-up.

### Opioid dose

The information about opioid use for symptom palliation (e.g., pain or dyspnea), both regular doses and the number of breakthrough doses per day from the initial consultation to the study endpoints, was collected by reviewing the patient’s medical records. The formulations and dosages of available opioids in Thailand are summarized in Appendix. The use of tramadol and methadone was excluded from the analysis due to their oral morphine equivalent not being reliably established [30]. The daily opioid dose for each type of opioid was then converted to an oral morphine equivalent (OME), following a standard ratio, as shown in Table 1 and 2.

**Table 1:** Equianalgesic dose (mg) of different types of opioids [47, 48]

Types of Opioids (Route)	Oral	Parenteral^a^
Morphine sulfate	30 mg	10 mg (IV), 15 mg (SC)
Pethidine hydrochloride (IV)	–	100 mg (IV)
Codeine phosphate tablet (Oral)	200 mg	–
Fentanyl citrate (IV)	–	0.1 mg
Methadone and Tramadol	Morphine dose equivalence not being reliably established	

^a^ *IV* intravenous route, *SC* subcutaneous route

**Table 2:** Equivalence between oral morphine and transdermal fentanyl [48, 49]

Transdermal fentanyl	Oral morphine
Fentanyl TTS 12 mcg/h	30 mg
Fentanyl TTS 25 mcg/h	60 mg
Fentanyl TTS 50 mcg/h	120 mg

The average daily opioid use of each patient during those specific periods was calculated by using the sum of the OME in that period divided by the number of days. For example, the average daily OME in the first week = Sum of OME in the first weeks divided by 7. Based on the previous data from our palliative program, which showed that most patients survived 2–3 months after the palliative consultation, we decided to calculate the average daily OME on a weekly basis for the first three months and then on a monthly basis until the study endpoints.

### Statistical analysis

The opioid doses were categorized into 2 groups: 1) ≤ 30 mg/day and 2) > 30 mg/day, based upon the recommended total daily starting dose for opioids in palliative care [31, 32]. The opioid dosages varied according to time, depending on the symptom severity and clinical conditions of the patients. Therefore, they were considered in the model as a time-dependent variable. The survival probability among the two categories of opioid doses was estimated using Kaplan–Meier curves and they were compared using the Log-rank test. The univariate Cox proportional hazards regression model was applied to assess the association between risk of death and different opioid doses, as well as other possible prognostic factors (i.e., age, sex, marital status, types of cancer, services, and concurrent cancer treatments, metastases, and baseline PPS and CCI). The multivariate Cox proportional hazards regression was applied to determine the independent association between those variables and risk of death. Only the factors that had a *P*-value less than 0.15 from the univariate model were considered in the multivariate Cox proportional hazards model.

All of the analyses were performed using STATA version 14. A *P*-value less than 0.05 was considered to be a statistically-significant level.

## Results

Four hundred and six patients received palliative care from the Department of Family Medicine, Ramathibodi Hospital, between January 2013 and December 2015. Eighty-nine patients that had non-cancer diagnoses were excluded from the analysis. The age of the study population ranged from 19 to 95 years, with a median of 63 years. Forty-eight point nine percent were female and only 11% had no evidence of metastases at the time of the palliative consultation. The major types of malignancies were gastrointestinal cancer (40.1%), primary lung cancer (18%), and head and neck cancer (13.6%). The median PPS level was 40% (ranging from 10% to 90%) and the median CCI score was 3 (ranging from 0 to 39). The most prescribed opioids in our study were morphine in different formulations and fentanyl patch. The median (IQR) of the OME among our patients was 6.43 mg/day (0.53, 27.36). Two of our patients did not receive any type of opioid during the study period. Thirteen patients (4.1%) received oral methadone during the study period. Seventy-two patients (22.7%) received oral tramadol and forty-nine patients (15.5%) received intravenous tramadol. The median survival of the patients was 33 days (ranging from 1 to 995 days). The details of the characteristics of our study population are depicted in Table 3.

**Table 3:** Characteristics of the patients (*N* = 317)

Characteristics	Number (%)
Age (median; range)	63 years (19–95)
Gender	
Female	155 (48.9)
Marital status	
• Married	220 (69.4)
• Widowed	43 (13.6)
• Single	31 (9.8)
• Divorced	23 (7.3)
Types of malignancies	
• Gastrointestinal cancer	127 (40.1)
• Primary lung cancer	57 (18)
• Head and neck cancer	43 (13.6)
• Genitourinary cancer	34 (10.7)
• Breast cancer	22 (6.9)
• Others	34 (10.7)
Metastases	
No evidence of metastases at initial consultation	35 (11)
PPS level (%)^a^	
• ≤ 30	130 (41)
• 40–60	102 (32.2)
• ≥ 70	65 (20.5)
Charlson comorbidity index	
• ≤ 3	196 (61.8)
• > 3	121 (38.2)
Types of service	
• Hospital admission	191 (60.3)
• Palliative care clinic visit	126 (39.7)
• Home visit	97 (30.6)
Concurrent cancer treatments	
• Chemotherapy	62 (19.6)
• Radiotherapy	49 (15.5)
• Surgery	13 (4.1)
Types of opioids^b^	
• Morphine sulfate (Oral)	235 (74.1)
• Morphine sulfate (Intravenous)	155 (48.9)
• Morphine sulfate (Subcutaneous)	5 (1.6)
• Fentanyl TTS 12 mcg/h	70 (22.1)
• Fentanyl TTS 25 mcg/h	78 (24.6)
• Fentanyl TTS 50 mcg/h	56 (17.7)
• Fentanyl citrate (Intravenous)	22 (6.9)
• Pethidine hydrochloride (Intravenous)	16 (5)
• Codeine phosphate (Oral)	24 (7.6)
Patient status at the last follow-up	
• Dead	228 (71.9)
• Alive	89 (28.1)

^a^ *N* = 297, missing values in 20 patients (6.3%)

^b^ *N* = 315, each patient could receive more than one type of opioid and two patients did not receive any opioid

The univariate Cox regression analysis revealed that the patients with PPS levels of 40 to 60% and greater than 70% had a higher chance of survival than patients with a PPS level of 30% and below 30%, with a hazard ratio of 0.27 (95%CI 0.20–0.38) and 0.11 (95%CI 0.07–0.16) respectively. Moreover, a palliative care clinic visit (HR 0.32, 95%CI 0.24–0.43), a home visit (HR 0.75, 95%CI 0.57–0.99), chemotherapy (HR 0.32, 95%CI 0.22–0.46), and radiotherapy (HR 0.53, 95%CI 0.36–0.78) were identified as factors that increased the probability of survival. On the other hand, the patients that needed hospital admission had a higher probability of dying (HR 2.03, 95%CI 1.54–2.67).

**Fig. 1:**
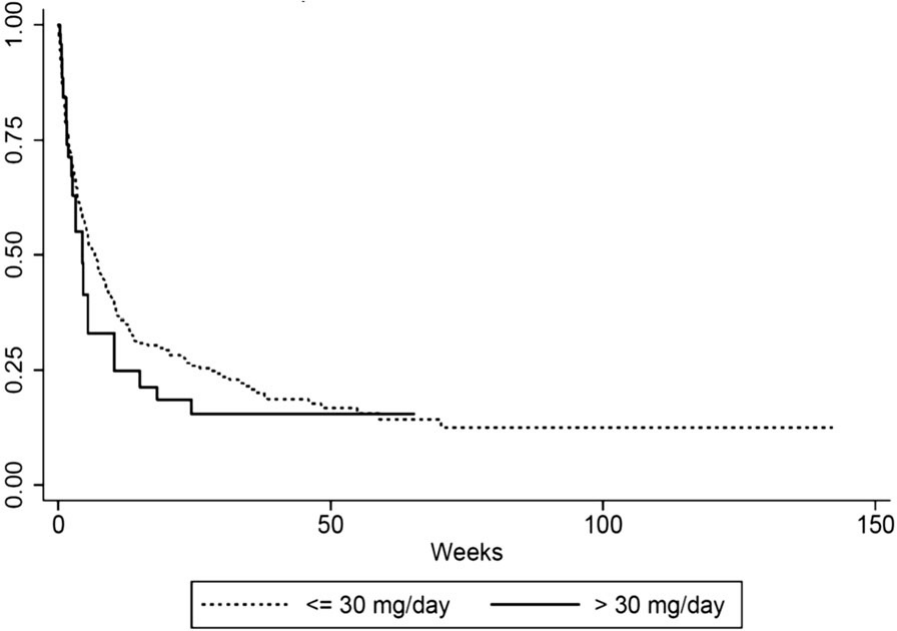
Survival curves of the study population categorized by oral morphine equivalent

We did not find any difference in the survival among the patients that received OME of ≤ 30 mg/day and more than 30 mg/day (HR 1.14, 95%CI 0.77–1.69). The median survival time for OME ≤ 30 and > 30 was 47 days and 31 days. The medians (IQR) of the OME in patients with OME ≤ 30 mg/day and > 30 mg/day were 3.08 (0.22, 9.37) and 80 (42.98, 148.74) respectively. The results of our analysis are shown in Table 4 and the survival curves of the two groups of patients receiving different opioid doses are shown in Fig. 1.

**Table 4:** Univariate Cox regression analysis of factors associated with survival

Factors	Number of deaths (%)	Hazard ratio	95%CI	*P*-value
Gender				
• Female	113 (72.9)	1.0	–	0.36
• Male	115 (71)	0.89	0.68,1.15	
Age				
• Age < 60	86 (68.3)	1.0	–	0.96
• Age ≥ 60	142 (74.4)	1.01	0.77,1.32	
Marital status				
• Married	153 (69.6)	1.0	–	0.62
• Divorced	19 (82.6)	1.30	0.81,2.10	
• Widowed	33 (76.7)	1.03	0.71,1.51	
• Single	23 (74.2)	1.23	0.80,1.91	
Type of malignancy				
• Head and neck cancer	26 (60.5)	1.0	–	0.26
• Breast cancer	16 (72.7)	1.40	0.75,2.62	
• Primary lung cancer	41 (71.9)	1.09	0.66,1.78	
• Gastrointestinal cancer	99 (78)	1.51	0.98,2.34	
• Genitourinary cancer	24 (70.6)	1.07	0.62,1.87	
• Others	22 (67.7)	1.15	0.65,2.03	
Metastases				
Evidence of metastases at consultation	204 (72.3)	1.0	–	0.33
No metastases	24 (68.5)	0.81	0.53,1.24	
PPS (%)				
• ≤ 30	113 (86.9)	1.0	–	<0.01
• 40–60	73 (71.6)	0.27	0.20,0.38	
• ≥ 70	30 (46.2)	0.11	0.07,0.16	
Type of service				
• No home visit	151 (68.6)	1.0	–	0.04
• Home visit	77 (79.4)	0.75	0.57,0.99	
• No palliative care clinic visit	154 (80.6)	1.0	–	<0.01
• Palliative care clinic visit	74 (58.7)	0.32	0.24,0.43	
• No hospital admission	81 (64.3)	1.0	–	<0.01
• Having hospital admission	147 (77)	2.03	1.54,2.67	
Concurrent treatment				
• No chemotherapy	195 (76.5)	1.0	–	<0.01
• Receiving chemotherapy	33 (53.2)	0.32	0.22,0.46	
• No radiotherapy	198 (73.9)	1.0	–	<0.01
• Receiving radiotherapy	30 (61.2)	0.53	0.36,0.78	
• No surgery	220 (72.4)	1.0	–	0.12
• Receiving surgery	8 (61.5)	0.60	0.29,1.20	
Charlson comorbidity index (CCI)				
• ≤ 3	132 (67.7)	1.0	–	0.26
• > 3	95 (78.5)	1.16	0.89,1.52	
Oral morphine equivalent (mg/day)				
• ≤ 30 mg/day	170 (69.7)	–	–	0.52
• > 30 mg/day	58 (79.5)	1.14	0.77,1.69

Previous preclinical studies have demonstrated that morphine may lead to cancer progression via many mechanisms in some specific types of cancer [21, 23]. Moreover, evidence from clinical practice has revealed that individuals respond differently to opioids, and that general differences between classes of opioids do exist [33, 34]. Therefore, we decided to perform a *post hoc* subgroup analysis of the association between different morphine doses and the survival of palliative care patients that had received only morphine. We found that a higher dose of morphine (>30 mg/day) was strongly associated with higher mortality (HR 4.09, 95%CI 1.89–8.78) with the median survival time for OME ≤ 30 and > 30 at 47 days and 31 days respectively. The multivariate Cox regression analysis, adjusted by PPS, concurrent chemotherapy and radiotherapy, palliative care clinic visit, hospital admission, and home visit, also revealed the same result (HR 4.13, 95%CI 1.83–9.31), as shown in Table 5.

**Table 5:** Cox regression analysis of the association between different morphine doses and the survival among palliative care patients that received morphine only (*N* = 118)

Morphine doses	Number of deaths^a^ (%)	Hazard ratio (95%CI)	*P*-value	Adjusted Hazard ratio^b^ (95%CI)	*P*-value
• ≤ 30 mg/day	82 (72.57)	–		–	
• > 30 mg/day	5 (100)	4.08 (1.89,8.78)	<0.01	4.13 (1.83, 9.31)	<0.01

^a^ *N* = 118

^b^Adjusted by PPS, chemotherapy, radiotherapy, palliative care clinic visit, hospital admission, and home visit

## Discussion

Concerns that opioids may hasten death can be a cause of physicians’ reluctance to prescribe or increase the dosage of opioids in palliative care settings, and could contribute to insufficient symptom palliation in the latest stage of cancer. The present study looked at the survival of cancer patients that received palliative care in various settings, e.g., outpatient, inpatient, and home care. We did not find any association between different opioid doses and the survival of the cancer patients in our palliative care program. This finding agrees with clinical experience and the findings of previous studies of palliative care populations [11–14, 16, 17, 35].

On the other hand, when we performed the *post hoc* subgroup analysis to find out if there was any association between different morphine doses and the survival among patients that received morphine only, we surprisingly found that a higher dose of morphine was associated with shorter survival period. This finding is consistent with the results from previous studies that showed the effects of morphine on cancer progression/recurrence and survival with some specific types of cancers, although the effects of morphine on these outcomes are still poorly understood [21, 25, 36, 37]. It is also worth mentioning that these effects have been explored in preclinical models using morphine as the archetypical opioid [38]. Nevertheless, the number of patients with OME > 30 mg/day in this subgroup analysis was very low and all died. This could be one limitation of the analysis and we believe that further, well-designed study is required to clarify if there is an association between classes of opioids and the survival of cancer patients. Moreover, there may be other potential confounding factors, such as the patients’ final symptom levels, that were not recorded in our study. Thus those patients needing a higher dose of morphine might have worse symptoms or at least need a higher dose to control them. A more advanced disease might mean increased levels of pain, necessitating higher doses of morphine. Therefore, it is too early to come to a conclusion concerning these effects of morphine in clinical practice.

The patients participating in our study had a median survival of 33 days, ranging from 1 to 995 days. This follow-up period was longer than that discussed in previous literature, where the follow-ups were generally short, over days or only a short number of weeks [39]. To the best of our knowledge, ours is the first study to analyze opioid use from the time of palliative consultation until the patient’s death by using the time-dependent covariate analysis in the Cox regression model, which represented the variation of actual opioid exposure better than measuring only the increased dose or the opioid dose used during the last days of life, as performed by other studies [14, 15, 18, 39, 40].

We also found that the higher Palliative Performance Scale, chemotherapy, and radiotherapy were among the factors strongly correlated with longer survival of the patients, which was consistent with previous reports [41–44]. Moreover, the patients that visited the palliative care clinic or had a home visit tended to survive longer, and the patients that needed hospital admission lived a shorter length of time in our study. This could be explained by the fact that only patients with good clinical or functional status survived long enough to receive those interventions, and those with complex conditions that needed hospital admission had a greater chance of dying earlier.

### Limitations

There are some limitations of our study worth mentioning. Since our study was a retrospective cohort study, the sample was not randomly determined. Other variables or residual confounders could have affected the differences between groups. It is worth mentioning that the doses of methadone and tramadol were not included in the analysis. Although, few patients received those opioids, this might have affected the results of the study to some extent. Most of the patients in our study were exposed to relatively low doses of opioids and this may have led to a “floor effect” where we may not have seen the significant difference in the survival among patients with different opioid doses in our study. Additionally, a patient’s survival is influenced by many complex factors that may not be measurable. For example, depression was identified as a factor associated with mortality among advanced cancer patients in a longitudinal study [45]. The effect of opioids on survival may be from better pain relief, which may interact with the patient’s psychological distress or depression. Moreover, most of our cancer patients survived for a few months after the palliative consultation. Therefore, the chronic effect (months to years) of opioids on survival was not adequately illustrated in this study. Further, there are wide differences in published opioid equianalgesic ratios and critical individual factors, such as gender differences, organ dysfunction, bidirectional differences of equivalence with certain opioids, drug interactions, and interindividual differences in pharmacokinetics and pharmacodynamics, which may impact equianalgesic doses [46]. Therefore, the calculated equianalgesic dose to oral morphine in this study may represent only the estimated opioid exposure and not the real-life exposure for each individual patient.

## Conclusion

Our study has demonstrated that the use of opioids of different doses in the palliative care population is not associated with shortened survival time. Based on our available data, we recommend that opioids be continued for pain control in patients with advanced cancer, as the ultimate goal of palliative care is to provide patients with the best quality of life during the trajectory of the illness. Nevertheless, future clinical research is required to clarify if there are any associations between different classes of opioids and the survival of cancer patients.

## Appendix

**Table 6:** Formulations and dosages of available opioids in Thailand [50]

Formulations	Available dosages
Tramadol hydrochloride (injectable form)	50 mg/ml
Tramadol hydrochloride (capsule)	50 mg
Tramadol hydrochloride (sustained release tablet)	100 mg
Morphine sulfate (injectable form)	10 mg/ml
Morphine sulfate (immediate release oral solution)	2 mg/ml
Morphine sulfate (immediate release tablet)	10 mg
MST Continus (sustained release morphine tablet)	10 mg, 30 mg, 60 mg
Kapanol (sustained release morphine capsule)	20 mg, 50 mg, 100 mg
Pethidine hydrochloride (injectable form)	50 mg/ml
Fentanyl GPO TTS^a^	12 mcg/h, 25 mcg/h, 50 mcg/h
Fentanyl citrate (injectable form)	0.1 mg/ml
Codeine phosphate (tablet)	15 mg, 30 mg
Methadone hydrochloride (tablet)	10 mg

^a^Manufactured by Hexal AG, Germany

## Abbreviations

IV: Intravenous route
OME: Oral morphine equivalent
SC: Subcutaneous route

## References

[1] WHO’s cancer pain ladder for adults: World Health Organization. Available from: http://www.who.int/cancer/palliative/painladder/en/. Accessed 1 June 2016.

[2] Lopez-Saca JM, Centeno C (2014). Opioids prescription for symptoms relief and the impact on respiratory function: updated evidence. Curr Opin Support Palliat Care.

[3] Reis-Pina P, Lawlor PG, Barbosa A (2015). Cancer-related pain management and the optimal use of opioids. Acta Med Port.

[4] Teno JM, Clarridge BR, Casey V, Welch LC, Wetle T, Shield R (2004). Family perspectives on end-of-life care at the last place of care. JAMA.

[5] Garcia-Toyos N, Escudero-Carretero MJ, Sanz-Amores R, Guerra-De Hoyos JA, Melchor-Rodriguez JM, Tamayo-Velazquez MI (2014). Preferences of caregivers and patients regarding opioid analgesic use in terminal care. Pain Med.

[6] Janssen DJ, de Hosson SM, bij de Vaate E, Mooren KJ, Baas AA (2015). Attitudes toward opioids for refractory dyspnea in COPD among Dutch chest physicians. Chron Respir Dis.

[7] Lin CC, Wang P, Lai YL, Lin CL, Tsai SL, Chen TT (2000). Identifying attitudinal barriers to family management of cancer pain in palliative care in Taiwan. Palliat Med.

[8] Rocker G, Young J, Donahue M, Farquhar M, Simpson C (2012). Perspectives of patients, family caregivers and physicians about the use of opioids for refractory dyspnea in advanced chronic obstructive pulmonary disease. CMAJ.

[9] Rurup ML, Borgsteede SD, van der Heide A, van der Maas PJ, Onwuteaka-Philipsen BD (2009). Trends in the use of opioids at the end of life and the expected effects on hastening death. J Pain Symptom Manage.

[10] Bercovitch M, Adunsky A (2004). Patterns of high-dose morphine use in a home-care hospice service: should we be afraid of it?. Cancer.

[11] Bengoechea I, Gutierrez SG, Vrotsou K, Onaindia MJ, Lopez JM (2010). Opioid use at the end of life and survival in a Hospital at Home unit. J Palliat Med.

[12] Azoulay D, Hammerman-Rozenberg R, Cialic R, Ein Mor E, Jacobs JM, Stessman J (2008). Increasing opioid therapy and survival in a hospice. J Am Geriatr Soc.

[13] Azoulay D, Jacobs JM, Cialic R, Mor EE, Stessman J (2011). Opioids, survival, and advanced cancer in the hospice setting. J Am Med Dir Assoc.

[14] Good PD, Ravenscroft PJ, Cavenagh J (2005). Effects of opioids and sedatives on survival in an Australian inpatient palliative care population. Intern Med J.

[15] Radha Krishna LK, Poulose JV, Tan BS, Goh C (2010). Opioid use amongst cancer patients at the end of life. Ann Acad Med Singapore.

[16] Alsirafy SA, Galal KM, Abou-Elela EN, Ibrahim NY, Farag DE, Hammad AM (2013). The use of opioids at the end-of-life and the survival of Egyptian palliative care patients with advanced cancer. Ann Palliat Med.

[17] Minami S, Fujimoto K, Ogata Y, Yamamoto S, Komuta K (2015). Opioids have no negative effect on the survival time of patients with advanced lung cancer in an acute care hospital. Support Care Cancer.

[18] Portenoy RK, Sibirceva U, Smout R, Horn S, Connor S, Blum RH (2006). Opioid use and survival at the end of life: a survey of a hospice population. J Pain Symptom Manage.

[19] Lin YL, Lin IC, Liou JC (2011). Symptom patterns of patients with head and neck cancer in a palliative care unit. J Palliat Med.

[20] van Hooft JE, Dijkgraaf MG, Timmer R, Siersema PD, Fockens P (2010). Independent predictors of survival in patients with incurable malignant gastric outlet obstruction: a multicenter prospective observational study. Scand J Gastroenterol.

[21] Singleton PA, Moss J, Karp DD, Atkins JT, Janku F (2015). The mu opioid receptor: a new target for cancer therapy?. Cancer.

[22] Zylla DM. Impact of opioid use on survival in patients with newly diagnosed stage IV non-hematologic malignancies. 2015 Palliative Care in Oncology Symposium; 2015.

[23] Nguyen J, Luk K, Vang D, Soto W, Vincent L, Robiner S (2014). Morphine stimulates cancer progression and mast cell activation and impairs survival in transgenic mice with breast cancer. Br J Anaesth.

[24] Skipworth RJ, Moses AG, Sangster K, Sturgeon CM, Voss AC, Fallon MT (2011). Interaction of gonadal status with systemic inflammation and opioid use in determining nutritional status and prognosis in advanced pancreatic cancer. Support Care Cancer.

[25] Halabi S, Lin CY, Kelly WK, Fizazi KS, Moul JW, Kaplan EB (2014). Updated prognostic model for predicting overall survival in first-line chemotherapy for patients with metastatic castration-resistant prostate cancer. J Clin Oncol.

[26] Boland JW, Ziegler L, Boland EG, McDermid K, Bennett MI (2015). Is regular systemic opioid analgesia associated with shorter survival in adult patients with cancer? A systematic literature review. Pain.

[27] Lopez-Saca JM, Guzman JL, Centeno C (2013). A systematic review of the influence of opioids on advanced cancer patient survival. Curr Opin Support Palliat Care.

[28] Charlson ME, Pompei P, Ales KL, MacKenzie CR (1987). A new method of classifying prognostic comorbidity in longitudinal studies: development and validation. J Chronic Dis.

[29] Chewaskulyong B, Sapinun L, Downing GM, Intaratat P, Lesperance M, Leautrakul S (2012). Reliability and validity of the Thai translation (Thai PPS Adult Suandok) of the Palliative Performance Scale (PPSv2). Palliat Med.

[30] Mercadante S, Caraceni A (2011). Conversion ratios for opioid switching in the treatment of cancer pain: a systematic review. Palliat Med.

[31] Klepstad P, Kaasa S, Borchgrevink PC (2011). Starting step III opioids for moderate to severe pain in cancer patients: dose titration: a systematic review. Palliat Med.

[32] NICE. Palliative care for adults: strong opioids for pain relief UK2016 [updated August 2016]. Available from: https://www.nice.org.uk/guidance/cg140/resources. Accessed 1 Sept 2016.

[33] Mercadante S, Bruera E (2016). Opioid switching in cancer pain: From the beginning to nowadays. Crit Rev Oncol Hematol.

[34] Naito T, Kawakami J (2015). Interindividual variation of pharmacokinetic disposition of and clinical responses to opioid analgesics in cancer pain patients. Yakugaku Zasshi.

[35] Thorns A, Sykes N (2000). Opioid use in last week of life and implications for end-of-life decision-making. Lancet.

[36] Lennon FE, Mirzapoiazova T, Mambetsariev B, Poroyko VA, Salgia R, Moss J (2014). The Mu opioid receptor promotes opioid and growth factor-induced proliferation, migration and Epithelial Mesenchymal Transition (EMT) in human lung cancer. PLoS One.

[37] Niu DG, Peng F, Zhang W, Guan Z, Zhao HD, Li JL (2015). Morphine promotes cancer stem cell properties, contributing to chemoresistance in breast cancer. Oncotarget.

[38] Sacerdote P, Manfredi B, Mantegazza P, Panerai AE (1997). Antinociceptive and immunosuppressive effects of opiate drugs: a structure-related activity study. Br J Pharmacol.

[39] Morita T, Tsunoda J, Inoue S, Chihara S (2001). Effects of high dose opioids and sedatives on survival in terminally ill cancer patients. J Pain Symptom Manage.

[40] Sykes N, Thorns A (2003). The use of opioids and sedatives at the end of life. Lancet Oncol.

[41] Kim AS, Youn CH, Ko HJ, Kim HM (2014). The survival time of terminal cancer patients: prediction based on clinical parameters and simple prognostic scores. J Palliat Care.

[42] Kristensen A, Vagnildhaug OM, Gronberg BH, Kaasa S, Laird B, Solheim TS (2016). Does chemotherapy improve health-related quality of life in advanced pancreatic cancer? A systematic review. Crit Rev Oncol Hematol.

[43] Mei AH, Jin WL, Hwang MK, Meng YC, Seng LC, Yaw WH (2013). Value of the Palliative Performance Scale in the prognostication of advanced cancer patients in a tertiary care setting. J Palliat Med.

[44] Myers J, Kim A, Flanagan J, Selby D (2015). Palliative performance scale and survival among outpatients with advanced cancer. Support Care Cancer.

[45] Lloyd-Williams M, Payne S, Reeve J, Dona RK (2014). Thoughts of self-harm and depression as prognostic factors in palliative care patients. J Affect Disord.

[46] Conversion Between Opioid Analgesics: A practical guide from the University of Alberta Multidisciplinary Pain Centre Faculty of Medicine & Dentistry, University of Alberta. Available from: https://www.ualberta.ca/medicine/institutes-centres-groups/multidisciplinary-pain-centre/for-healthcareprofessionals/opioid-conversion-guide. Accessed 1 June 2016.

[47] Canadian Guideline for Safe and Effective Use of Opioids for Chronic Non-Cancer Pain: Michael G. DeGroote National Pain Centre 2010. Available from: http://nationalpaincentre.mcmaster.ca/opioid/index.html. Accessed 1 June 2016.

[48] Choosing and Changing Opioids: Scottish Palliative Care Guidelines; 2014. Available from: http://www.palliativecareguidelines.scot.nhs.uk/guidelines/pain/choosing-and-changing-opioids.aspx. Accessed 1 June 2016.

[49] Breitbart W, Chandler S, Eagel B, Ellison N, Enck RE, Lefkowitz M (2000). An alternative algorithm for dosing transdermal fentanyl for cancer-related pain. Oncology (Williston Park).

[50] National List of Essential Medicines: Food and Drug Administration Thailand, Ministry of Public Health. Available from: http://drug.fda.moph.go.th:81/nlem.in.th/medicine/essential/list/73.

